# Quantitative analysis of retinal and choroidal microvascular changes in patients with diabetes

**DOI:** 10.1038/s41598-018-30699-w

**Published:** 2018-08-14

**Authors:** Mirinae Kim, Seung Yong Choi, Young-Hoon Park

**Affiliations:** 1 Department of Ophthalmology and Visual Science, College of Medicine, The Catholic University of Korea, Seoul, Republic of Korea; 2 Catholic Institute for Visual Science, College of Medicine, The Catholic University of Korea, Seoul, Korea

## Abstract

The relationship between choroidal and retinal microvascular changes has not yet been well described, and there were limited data on diagnostic ability of optical coherence tomography (OCT)-derived vascular parameters for determining diabetic retinopathy (DR) progression. We quantitatively analyzed OCT-derived vascular parameters at superficial (SCP) and deep retinal capillary plexus (DCP), and choroid. We assessed foveal avascular zone (FAZ), vessel density, vessel length density, and choroidal vascularity index in conjunction with DR stage. In this study, patients with diabetes and healthy controls were retrospectively analyzed. One-hundred seventy-four eyes were divided into six groups as follows: Healthy controls, no DR, mild non-proliferative DR (NPDR), moderate NPDR, severe NPDR, and proliferative DR. There were significant quantitative changes in retinal and choroidal vascular parameters with DR progression. The FAZ area and perimeter correlated positively with worsening DR severity; the FAZ circularity index, retinal vessel density, retinal vessel length density, and choroidal vascularity index correlated negatively with worsening severity. Among these, FAZ circulatory index demonstrated good diagnostic performance for DR. Our results cautiously suggest that functional circulatory disturbances in retinal and choroidal vasculatures occur before DR presents. As DR progresses, DCP retinal microvasculature changes precede SCP changes.

## Introduction

Optical coherence tomography (OCT) angiography is a noninvasive, dye-free imaging modality that can evaluate the retinal microvasculature by capturing the dynamic motion of erythrocytes^1^. OCT angiography has many advantages over conventional fluorescein angiography (FA). It can optically dissect and visualize flows in various layers of the retina and obtain high-resolution images^1^. At present, the gold standard in the diagnosis and classification of diabetic retinopathy (DR) is FA^2^. However, FA cannot directly image the retinal microvasculature layer by layer or detect subtle microvascular changes, especially in early DR.

The literature includes several studies that used OCT angiography on patients with DR^3–7^. In patients with diabetes mellitus (DM), studies reported modifications of the foveal avascular zone (FAZ), perfusion density, and fractal dimension (vascular complexity). Increased FAZ area, decreased perfusion density, and increased fractal dimension were associated with worsening DR severity. Previously, we used OCT to evaluate the choroidal changes in patients with DM, and we concluded that the diabetic choroidopathy precedes the retinopathy^8^. However, the relationship between choroidal and retinal vascular changes has not yet been described. There were limited data analyzing OCT-derived vascular parameters to evaluate diabetic retinopathy and choroidopathy together. Moreover, there are few data on the diagnostic abilities of OCT-derived vascular parameters.

The purpose of our study was to quantitatively assess retinal and choroidal microvascular changes in patients with DM in conjunction with DR stage and to evaluate diagnostic ability for determining DR progression. Based on the results, we attempted to understand the pathogenesis and sequence of retinal and choroidal vascular changes in patients with DM.

## Results

### Demographic and clinical characteristics

We included a total of 174 eyes in this study (132 eyes of 81 patients with type 2 DM and 42 eyes of 28 healthy controls). We divided eyes into six groups as follows: healthy controls (group 1, *n* = 42), no DR (group 2, *n* = 30), mild non-proliferative DR (NPDR) (group 3, *n* = 22), moderate NPDR (group 4, *n* = 23), severe NPDR (group 5, *n* = 42), and proliferative DR (PDR) (group 6, *n* = 15). We summarized the demographic, ocular, and systemic characteristics of the subjects in Table 1. Of the 132 diabetic eyes, 98 were from women and 76 were from men. The mean age was 57.2 ± 13.0 years and the mean duration of DM was 10.8 ± 7.4 years. There were no significant differences in age, sex, refractive error, visual acuity, intraocular pressure, disease duration, or glycated hemoglobin (HbA1c) level. Fasting blood glucose level (*P* < 0.001), systolic blood pressure (*P* = 0.012), and diastolic blood pressure (*P* = 0.034) differed significantly between each DR group. The fasting blood glucose level was significantly higher in the severe NPDR group (*P* = 0.044). Systolic blood pressure was significantly higher in the moderate NPDR (*P* = 0.019) and severe NPDR groups (*P* = 0.016).

**Table 1:** Demographic and clinical characteristics of the eyes (n = 174) according to the study group

Variables	Healthy controls (n = 42)	No DR (n = 30)	Mild NPDR (n = 22)	Moderate NPDR (n = 23)	Severe NPDR (n = 42)	PDR (n = 15)	*P* value
No. of eyes	42	30	22	23	42	15	
Age, years	51.7 ± 14.0	55.9 ± 13.5	60.0 ± 13.6	58.7 ± 12.9	57.6 ± 10.4	57.6 ± 9.9	0.118
Sex, n (%)							0.330
Female	30 (71.4)	17 (56.7)	11 (50.0)	11 (47.8)	22 (52.4)	7 (46.7)	
Male	12 (28.6)	13 (43.3)	11 (50.0)	12 (52.2)	20 (47.6)	8 (53.3)	
Years with diabetes	—	5.5 ± 4.5	11.7 ± 8.3	13.9 ± 9.6	11.5 ± 5.0	11.9 ± 6.9	0.411
HbA1c,%	—	6.7 ± 0.9	7.2 ± 1.4	7.1 ± 0.7	7.6 ± 1.3	7.0 ± 0.5	0.253
Fasting blood sugar, mg/dL	—	144.6 ± 60.6	154.1 ± 52.4	130.3 ± 20.5	168.9 ± 51.5	138.5 ± 35.9	**<0**.**001**
Systolic BP, mmHg	—	120.7 ± 13.0	124.5 ± 10.9	129.2 ± 16.5	128.3 ± 12.3	128.0 ± 7.6	**0**.**012**
Diastolic BP, mmHg	—	76.2 ± 9.4	78.6 ± 8.2	74.5 ± 8.3	76.4 ± 10.9	76.1 ± 9.2	**0**.**034**
**Ophthalmologic examination**							
BCVA, logMAR	0.06 ± 0.10	0.06 ± 0.10	0.05 ± 0.08	0.06 ± 0.10	0.07 ± 0.11	0.12 ± 0.12	0.224
SE, diopter	−0.89 ± 2.00	−0.48 ± 1.39	0.11 ± 1.22	−0.33 ± 1.55	−0.70 ± 1.43	−0.67 ± 1.50	0.758
IOP, mmHg	14.6 ± 2.9	15.7 ± 3.6	15.0 ± 3.3	14.3 ± 2.2	15.6 ± 2.8	15.7 ± 4.0	0.083

Data are expressed as mean ± standard deviation (95% confidence interval).

Statistical tests: an analysis of variance (ANOVA) (continuous data); the Cochran-Armitage test (categorical data, e.g. sex) for trends; the Kruskal-Wallis test for visual acuity.

Factors with statistical significance are shown in bold.

BCVA, best-corrected visual acuity; BP, blood pressure; DR, diabetic retinopathy; IOP, intraocular pressure; logMAR, logarithm of the minimum angle of resolution; NPDR, non-proliferative diabetic retinopathy; PDR, proliferative diabetic retinopathy; SE, spherical equivalent.

### OCT-derived vascular parameters

Table 2 displays the OCT-derived retinal and choroidal vascular parameters. It shows that the FAZ area and FAZ perimeter correlated positively with worsening DR severity levels. In contrast, the FAZ circularity index, retinal vessel density, retinal vessel length density, and choroidal vascularity index (CVI) correlated negatively with worsening DR severity levels.

**Table 2:** Vascular parameters at the superficial and deep retinal capillary plexuses and the choroidal vasculature.

Parameters	Healthy controls (n = 42)	No DR (n = 30)	Mild NPDR (n = 22)	Moderate NPDR (n = 23)	Severe NPDR (n = 42)	PDR (n = 15)	adjusted β coefficient* ± SE	adjusted *P* value*
Superficial retinal capillary plexus								
FAZ area (mm^2^)	0.40 ± 0.13	0.42 ± 0.10	0.41 ± 0.10	0.42 ± 0.11	0.47 ± 0.10	0.50 ± 0.17	0.03 ± 0.01	**0**.**001**
FAZ perimeter (mm)	2.54 ± 0.42	2.89 ± 0.42	3.05 ± 0.40	3.13 ± 0.49	3.34 ± 0.48	3.42 ± 0.54	0.14 ± 0.03	**<0**.**001**
FAZ circulatory index	0.77 ± 0.14	0.62 ± 0.08	0.55 ± 0.07	0.54 ± 0.06	0.54 ± 0.10	0.51 ± 0.09	−0.02 ± 0.01	**0**.**009**
Vessel density (%)	35.95 ± 0.59	35.90 ± 0.81	35.73 ± 0.85	35.14 ± 0.79	35.27 ± 0.84	34.77 ± 0.70	−0.25 ± 0.06	**<0**.**001**
Vessel length density (mm^−1^)	15.75 ± 2.93	14.50 ± 3.49	13.32 ± 3.46	12.51 ± 3.25	13.37 ± 2.64	13.38 ± 2.86	−0.24 ± 0.23	0.313
Deep retinal capillary plexus								
FAZ area (mm^2^)	0.52 ± 0.14	0.55 ± 0.18	0.60 ± 0.22	0.63 ± 0.21	0.60 ± 0.15	0.64 ± 0.19	0.02 ± 0.01	0.142
FAZ perimeter (mm)	2.87 ± 0.53	3.27 ± 0.69	3.80 ± 0.73	3.69 ± 0.73	3.80 ± 0.06	4.01 ± 0.65	0.15 ± 0.05	**0**.**006**
FAZ circulatory index	0.79 ± 0.15	0.65 ± 0.10	0.52 ± 0.07	0.53 ± 0.09	0.53 ± 0.09	0.51 ± 0.08	−0.03 ± 0.01	**<0**.**001**
Vessel density (%)	34.95 ± 0.72	35.03 ± 0.75	34.94 ± 0.61	34.72 ± 0.87	34.70 ± 0.98	34.32 ± 0.96	−0.15 ± 0.06	**0**.**022**
Vessel length density (mm^−1^)	18.78 ± 2.32	18.42 ± 2.59	16.89 ± 3.04	16.80 ± 2.56	16.48 ± 1.88	16.83 ± 2.53	−0.39 ± 0.18	**0**.**043**
Choroidal vascularity index (%)	69.21 ± 2.24	67.06 ± 3.98	66.60 ± 3.03	66.18 ± 3.04	66.15 ± 2.63	63.10 ± 3.45	−0.68 ± 0.25	**0**.**009**

Data are expressed as mean ± standard deviation (95% confidence interval).

Linear mixed model.

Factors with statistical significance are shown in bold.

*Adjusted for age, sex, and diastolic blood pressure.

DR, diabetic retinopathy; FAZ, foveal avascular zone; NPDR, non-proliferative diabetic retinopathy; PDR, proliferative diabetic retinopathy.

We also assessed the influence of potential confounders in the vascular parameters. In the linear regression analysis, diastolic blood pressure was significantly associated with the FAZ area, retinal vessel density, and retinal vessel length density at the superficial (SCP) and deep capillary plexus (DCP) (all *P* < 0.05). For this reason, we evaluated the vascular parameters after adjusting for age, sex, and diastolic blood pressure.

As shown in Table 2, the mean FAZ circulatory indexes at the SCP in groups 1–6 were 0.77 ± 0.14, 0.62 ± 0.08, 0.55 ± 0.07, 0.54 ± 0.06, 0.54 ± 0.10, and 0.51 ± 0.09, respectively (adjusted β coefficient ± the standard error [SE] = −0.02 ± 0.01, *P* = 0.009). We observed a similar trend at the DCP, with mean FAZ circulatory indexes of 0.79 ± 0.15, 0.65 ± 0.10, 0.52 ± 0.07, 0.53 ± 0.09, 0.53 ± 0.09, and 0.51 ± 0.08 in groups 1–6, respectively (adjusted β coefficient ± SE = −0.03 ± 0.01, *P* < 0.001).

**Figure 1:**
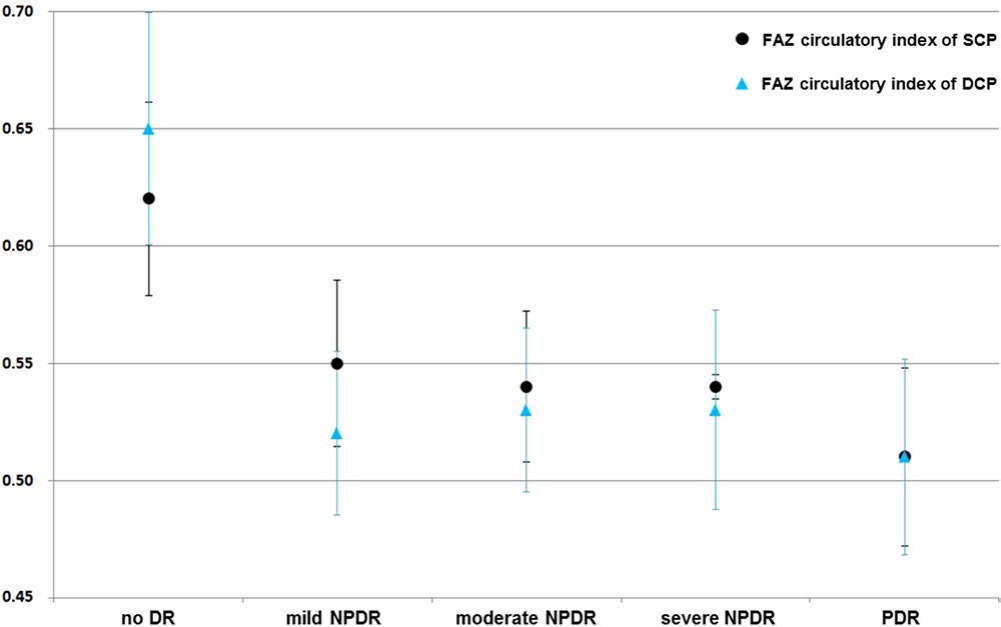
The graph demonstrates the foveal avascular zone (FAZ) circulatory indexes at the superficial capillary plexus (SCP) and deep capillary plexus (DCP) in diabetic patients. In the early stages of diabetic retinopathy (DR), the decreasing trend of the FAZ circulatory index is remarkable at the DCP. Notes: DR, diabetic retinopathy; NPDR, non-proliferative diabetic retinopathy; PDR, proliferative diabetic retinopathy.

Figure 1 shows the FAZ circularity index at the SCP and DCP with increasing DR severity in diabetic patients. In the early stages of DR, the decreasing trend in the FAZ circulatory index was remarkable at the DCP.

### ROC curve analysis of the vascular parameters

Tables 3 and 4 summarize the results of receiver operating characteristic (ROC) curve analyses of OCT-derived vascular parameters. The highest areas under the curve (AUC) were similar to those of the FAZ circulatory indexes at the SCP (AUC = 0.896; 95% CI, 0.831–0.962) and DCP (AUC = 0.898; 95% CI, 0.834–0.962) in discriminating no DR from other DR stages. We also observed the highest AUCs for discriminating early DR from advanced DR (including severe NPDR and PDR) in the FAZ circulatory index at the SCP (AUC = 0.773; 95% CI, 0.694–0.853) and DCP (AUC = 0.764; 95% CI, 0.679–0.848). The CVI achieved relatively high specificity (97.6%; 95% CI, 87.7–99.6%) in discriminating no DR from other DR stages. However, the sensitivity and specificity of the CVI (76.3% and 57.4%, respectively) fell to low levels in discriminating early DR from advanced DR.

**Table 3:** ROC curve analysis of vascular parameters between eyes without DR and eyes with other DR stages.

Parameters	AUC (95% CI)	Cutoff point^*^	Sensitivity (95% CI)	Specificity (95% CI)
Superficial retinal capillary plexus				
FAZ circulatory index	0.896 (0.831–0.962)	0.66	85.6% (78.6–90.6%)	83.3% (69.4–91.7%)
Vessel density (%)	0.684 (0.585–0.782)	35.42	47.0% (38.7–55.5%)	88.1% (75.0–94.8%)
Vessel length density (mm^−1^)	0.711 (0.599–0.823)	16.48	78.8% (71.1–84.9%)	59.5% (44.5–73.0%)
Deep retinal capillary plexus				
FAZ circulatory index	0.898 (0.834–0.962)	0.66	84.1% (76.9–89.4%)	83.3% (69.4–91.7%)
Vessel density (%)	0.553 (0.450–0.656)	34.58	37.1% (29.4–45.6%)	78.6% (64.1–88.3%)
Vessel length density (mm^−1^)	0.695 (0.582–0.807)	19.44	81.1% (73.5–86.8%)	52.4% (37.7–66.6%)
Choroidal vascularity index (%)	0.788 (0.715–0.861)	66.27	53.0% (44.6–61.3%)	97.6% (87.7–99.6%)

*Cutoff point based on the Youden Index.

AUC, area under the curve; CI, confidence interval; DR, diabetic retinopathy; FAZ, foveal avascular zone; ROC curve, receiver operating characteristic curve.

**Table 4:** ROC curve analysis of the vascular parameters between early and advanced DR stages.

Parameters	AUC (95% CI)	Cutoff point*	Sensitivity (95% CI)	Specificity (95% CI)
Superficial retinal capillary plexus				
FAZ circulatory index	0.773 (0.694–0.853)	0.62	81.3% (71.3–88.3%)	62.8% (52.7–71.9%)
Vessel density (%)	0.752 (0.672–0.832)	35.21	52.5% (41.7–63.1%)	86.2% (77.8–91.7%)
Vessel length density (mm^−1^)	0.656 (0.559–0.754)	16.48	87.5% (78.5–93.1%)	45.7% (36.0–55.8%)
Deep retinal capillary plexus				
FAZ circulatory index	0.764 (0.679–0.848)	0.66	92.5% (84.6–96.5%)	51.1% (41.1–60.9%)
Vessel density (%)	0.610 (0.509–0.711)	34.31	40.0% (30.0–51.0%)	87.2% (79.0–92.5%)
Vessel length density (mm^−1^)	0.691 (0.596–0.784)	19.25	88.8% (80.0–94.0%)	45.7% (36.0–55.8%)
Choroidal vascularity index (%)	0.704 (0.618–0.791)	67.46	76.3% (65.9–84.2%)	57.4% (47.4–67.0%)

Early DR include no DR, mild NPDR, and moderate NPDR, and advanced DR include severe NPDR and PDR group.

*Cutoff point based on the Youden Index.

AUC, area under the curve; CI, confidence interval; DR, diabetic retinopathy; FAZ, foveal avascular zone; NPDR, non-proliferative diabetic retinopathy; PDR, proliferative diabetic retinopathy; ROC curve, receiver operating characteristic curve.

## Discussion

In accordance with the literature, our study demonstrated that there are significant quantitative changes in retinal and choroidal vascular parameters in conjunction with DR progression. The FAZ area and FAZ perimeter correlated positively with worsening DR severity levels. In contrast, the FAZ circularity index, retinal vessel density, retinal vessel length density, and CVI correlated negatively with worsening DR severity levels. Furthermore, these changes in vascular parameters were evident even in diabetic patients without DR.

Enlargement of the FAZ area can indicate the microcirculatory status of the central fovea. Previous studies reported enlargement and distortion of the FAZ in patients with DM regardless of the presence of DR^5^. In accordance with the literature, the FAZ area increased and the FAZ circularity index decreased as DR progressed in our study populations. Among the FAZ-related parameters, the FAZ circulatory index at the DCP showed the highest diagnostic ability in discriminating no DR from other DR stages. The FAZ circulatory index at the SCP showed the highest diagnostic ability in discriminating early DR from advanced DR. Someone might call the reproducibility and repeatability of the manually measured FAZ into question. Several studies presented good reproducibility and repeatability of manually outlined and measured FAZ-related parameters in normal eyes and diabetic eyes^9–12^. One previous study reported that FAZ alterations at the DCP might be subject to greater inter-observer variability in normal populations^11^. The high variability of FAZ measurements cast doubt on the reliability of FAZ-related parameters as a diagnostic tool in clinical practice, thus requiring further studies.

Retinal vascular changes including vascular tortuosity, caliber, fractal dimensions, capillary dropout, branching angle have correlation with diabetes^13,14^. In our study, we calculated both of the retinal vessel density and vessel length density. The difference between the two is that, retinal vessel density includes the concept of the thickness of microvessels. The retinal vessel length density showed higher diagnostic ability in discriminating no DR from other DR stages. Our data carefully provide an evidence that the architectural change of retinal microvasculature is more important than the thickness of microvessels in early DR.

We carefully suggest that changes in the retinal capillary plexus first occur at the DCP in the early stages of DR. In the advanced stages of DR, changes in the retinal capillary plexus appear prominently at the SCP. Previous studies demonstrated that the density of the smaller vessels in the deep retinal vascular network was greater than that in the superficial network^15,16^. Given the association between vascular parameters and diastolic blood pressure, relatively low blood flow might have a greater impact on the DCP, leading to hypoxia. Further, anatomically speaking, the SCP connects directly to retinal arterioles with a higher perfusion pressure and oxygen supply, so it can be better preserved from hypoxic damage in retinal vascular diseases including DM and retinal vein occlusion^17^.

In our previous study, we evaluated the choroidal vasculature in patients with DR^8^. We concluded that the ischemic changes in the choroidal vasculature could be the primary event in DM even where there is no DR. Some previous reports support our hypothesis; Muir *et al*.^18^ suggested that a choroidal blood flow deficit can be an early pathologic change in DR in an animal model. The size and density of choroidal vessels reduced with DM progression^19^ and choroidal blood flow reduced in patients with DM before the manifestation of retinopathy^20^.

From these findings, we cautiously suggest that a change in the choroidal vasculature is the primary event in patients with DM; then, retinal microvascular changes appear first at the DCP and later at the SCP. There is evidence of these microvascular disturbances even if there is no evidence of DR on a conventional imaging study.

Our study had some limitations. First, as is widely known, OCT angiography has issues regarding various artifacts, and artifacts appear more frequently in eyes with poor vision and retinal diseases^21,22^. In this study, we excluded OCT angiography images with poor image quality or diabetic macular edema (DME) and this might introduce selection bias. Much improvement is still necessary to assess vascular parameters in patients with concurrent DR and DME in clinical practice. Second, FAZ area and retinal vessel density can be influenced by spherical equivalent or axial length^23,24^. Further, we used the Niblack auto local threshold technique in binarizing the OCT images. It might be a source of error since this technique has not been validated so far. Third, we only included 15 patients with PDR. This was because we only included treatment-naïve patients and excluded cases of PDR with DME or media opacity such as a vitreous hemorrhage. Fourth, we only analyzed a 3-mm × 3-mm OCT angiography scan based on the work of Ho *et al*.^25^. They reported that a 3-mm × 3-mm scan delineated the FAZ more clearly than a 6-mm × 6-mm scan. However, a previous study suggested that early diabetic changes might occur in the mid-peripheral retina^26^. For this reason, a larger area might provide more representative information on retinal microvascular changes. In addition, each image was graded by a single vitreoretinal specialist only. Some readers might call into question about the inter-grader repeatability. Future studies should address these limitations.

In summary, before there is evidence of DR, there are functional circulatory disturbances both in the retinal and choroidal vasculatures. OCT-derived vascular parameters including the FAZ circulatory index, retinal vessel density, retinal vessel length density, and the CVI could assess the progression of DR. Among these, the FAZ circulatory index showed good diagnostic performance for DR. Our results cautiously suggest that changes in the choroidal vasculature could be the primary event in diabetic eyes, even where there is no DR. As DR progresses, changes in the retinal microvasculature at the DCP precede changes at the SCP. These results will aid in understanding the pathogenesis of DR. Further studies are necessary to support these findings and correlate them with morphological and functional parameters.

## Methods

### Study population

The study included patients with a confirmed diagnosis of type 2 DM and healthy controls. We recruited all participants between December 2016 and December 2017 at Seoul St. Mary’s Hospital in Korea and conducted a retrospective chart review. This retrospective cohort study adhered to the tenets of the Declaration of Helsinki. Institutional Review Board (IRB)/Ethics Committee approval was obtained from the Catholic University of Korea, which waived the requirement for obtaining informed patient consent because of the retrospective nature of the study.

Exclusion criteria were as follows: (1) refractive errors greater than ±6 diopters (as the spherical equivalent), (2) eyes with a history of any ocular trauma, laser treatment, or intraocular surgery, (3) eyes with a history of intravitreal or sub-Tenon’s injections, (4) other systemic disease that could affect the eye, (5) the presence of other retinal diseases, including glaucoma, age-related macular degeneration, retinal vein occlusion, epiretinal membrane, or neurodegenerative disease, (6) macular edema or media opacity that could affect image quality, (7) pachychoroid pigment epitheliopathy in either of the subject’s eyes (such as central serous chorioretinopathy or polypoidal choroidal vasculopathy)^27^, and (8) any history of uveitis.

We recruited healthy subjects from consecutive patients scheduled for a routine ocular examination and refractive error correction at Seoul St. Mary’s Hospital in Korea. We applied the same exclusion criteria.

We recorded demographic information and comprehensive medical and ophthalmologic histories at the initial visit. All subjects underwent ocular examinations including best-corrected visual acuity (BCVA) evaluation (logarithm of the minimum angle of resolution scale [logMAR]), non-contact pneumatic tonometry, slit-lamp biomicroscopy, dilated fundus examination, and OCT.

### DR grading

We classified the DR grade as no DR, mild NPDR, moderate NPDR, severe NPDR, or PDR according to the modified Early Treatment Diabetic Retinopathy Study (ETDRS) retinopathy severity scale^28^. Patients suspected of severe NPDR or PDR underwent FA. Each image was graded by an experienced vitreoretinal specialist; in total three specialists contributed for the grading in the study. The DR grading and the quantitative analysis of OCT images were performed in a double-blind manner.

### SS-OCT and OCT angiography image acquisition

During the same visit, all study subjects underwent swept-source (SS)-OCT examination (DRI Triton, Topcon, Tokyo, Japan), which contains a 1,050-nm-wavelength swept light source and has a scanning speed of 100,000 A-scans/second. We obtained a six-line radial pattern scan (1,024 A-scans) centered on the fovea from each eye. We also acquired OCT angiography volume scans with 3-mm × 3-mm images of the macula centered on the fovea. The OCT device automatically segments the layers using a built-in segmentation algorithm for the superficial plexus (2.6 μm below the internal limiting membrane to 15.6 μm below the junction between the inner plexiform and inner nuclear layers (IPL/INL) and deep plexus (15.6 μm below the IPL/INL to 70.2 μm below the IPL/INL).

*En face* projections of volumetric scans allow for the visualization of structural and vascular details within segmented retinal layer boundaries^29,30^. We only used OCT images with a signal strength index >60 and excluded scans with poor image quality. Scans with poor image quality met these criteria: (1) poor fixation resulting in a double vessel pattern and motion artifacts, (2) weak local signal or poor clarity, (3) macular edema, and (4) macular segmentation errors. Two vitreoretinal specialists evaluated the quality of each image independently.

### Assessment of the foveal avascular zone

**Figure 2:**
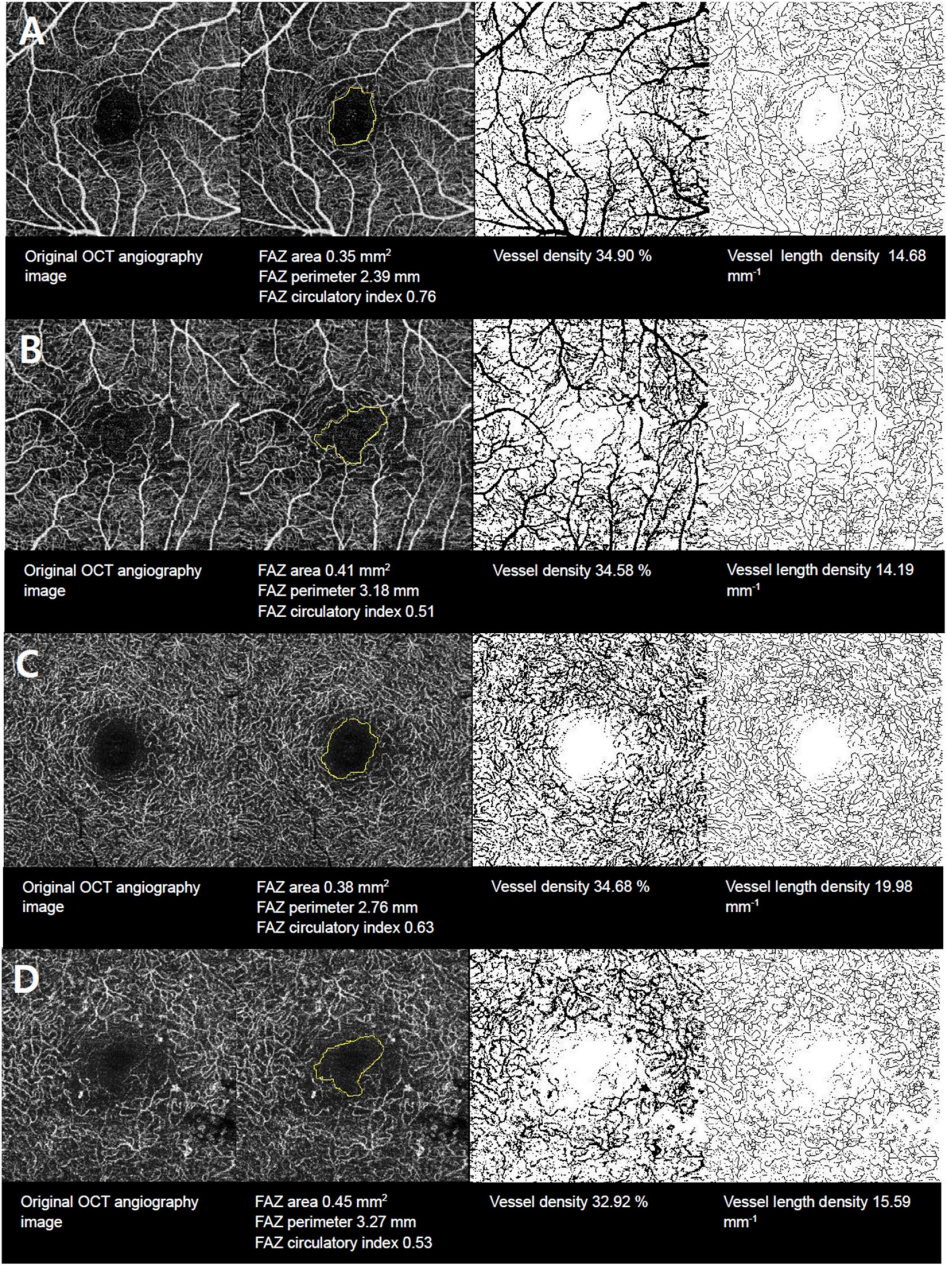
Representative *en face* optical coherence tomography(OCT) angiography images demonstrate visualization of (**A**) the superficial retinal capillary plexus with no diabetic retinopathy (DR), (**B**) the superficial retinal capillary plexus in proliferative DR (PDR), (**C**) the deep retinal capillary plexus with no DR, and (**D**) the deep retinal capillary plexus in PDR. Notes: FAZ, foveal avascular zone.

We performed a quantitative analysis of OCT angiography-derived images including FAZ area, FAZ perimeter, retinal vessel density, and retinal vessel length density measurements using ImageJ software (version 1.51; https://imagej.nih.gov/ij/; National Institutes of Health, Bethesda, MD, USA). The quantitative analysis of images and the DR grading process were performed by independent observers in a double-blind manner. OCT images of the participants were anonymized before being assessed by a masked observer. Figure 2 shows a representative depiction of the image processing used to obtain vascular parameters.

We outlined the FAZ area and perimeter manually along the innermost capillaries on *en face* OCT angiography images at the SCP and DCP. We set the image scale using a known image size of 320 × 320 pixels. Because we used an image 3 mm × 3 mm in size, the pixel aspect ratio was 1.0, resulting in a scale of 106.67 pixels per millimeter. With this image scale, we measured the FAZ area and perimeter manually. We measured the FAZ circularity index using the following equation: FAZ circularity index = 4π × area/(perimeter)^2^. This circularity index is a measure of the compactness of a shape relative to a circle; a ratio closer to one indicates a circular shape and a ratio closer to zero indicates an irregular shape^31^.

### Assessment of retinal vessel density and vessel length density

We measured retinal vessel density and vessel length density at the SCP and DCP. Using the Niblack auto local threshold technique, we binarized the images of each of the plexuses to convert them from grayscale into black-and-white images. We calculated vessel density as the percentage of the area occupied by blood vessels in the selected region. To measure the vessel length density, we skeletonized the binarized OCT angiography images to show the blood vessels as 1-pixel-wide lines and used ImageJ to count the numbers of black pixels and total pixels^32^.

### Assessment of the choroidal vascularity index

To quantitatively analyze the choroidal vasculature, we used the CVI. To assess the CVI, we selected the raster scan passing through the fovea on the SS-OCT device for image binarization. We segmented it using the protocol described by Agrawal *et al*.^33^ and in our previous study^8^. We measured the CVI in the subfoveal region within a width of 1,500 μm (750 μm on either side of the fovea). Using the polygon selection tool, we selected the total choroidal area (TCA) for the subfoveal region and added regions of interest (ROIs) to the ROI manager. After converting the image to an 8-bit image, we applied a Niblack auto local threshold tool. We highlighted the luminal area (LA) by applying a color threshold, which we subsequently added to the ROI manager. To determine the LA within the initially selected polygon, we selected and merged both areas in the ROI manager via an “AND” operation. We added this composite third area to the ROI manager. The first area represented the entirety of the selected choroid or TCA; the third composite area represented the vascular area or LA. We defined the CVI as the ratio of the LA to the TCA.

### Statistical analys  

We conducted an exploratory analysis of all variables. We expressed categorical data as absolute numbers and continuous data as the mean ± the standard deviation (SD) (95% confidence interval [CI]). We performed all statistical analyses using statistical software (SAS version 9.3; SAS Institute Inc., Cary, NC, USA).

We used a one-way analysis of variance (ANOVA) or the Cochran-Armitage test for clinical characteristic trends according to the DR group. When we used data from both eyes of the same patient, we calculated two-tailed *P* values using the linear mixed model approach and regarded the eye as the unit of analysis. We considered *P*-values < 0.05 as significant. We determined associations between vascular parameters (dependent variables) and ocular and systemic factors (independent variables) using a linear regression analysis.

To investigate the diagnostic ability of the vascular parameters to differentiate DR from no DR and early DR from advanced DR (including severe NPDR and PDR), we calculated the areas under the receiver operating characteristic curves. Greater AUCs indicate greater diagnostic ability. We calculated the specificities and sensitivities of the vascular parameters with cut-off values generated from the Youden index (the point with the optimal balance of specificity and sensitivity)^34^.

## Data Availability

The datasets during and/or analyzed during the current study are available from the corresponding author on reasonable request.

## References

[1] Spaide RF, Klancnik JM, Cooney MJ (2015). Retinal vascular layers imaged by fluorescein angiography and optical coherence tomography angiography. JAMA ophthalmology.

[2] Fluorescein angiographic risk factors for progression of diabetic retinopathy. ETDRS report number 13. Early Treatment Diabetic Retinopathy Study Research Group. *Ophthalmology* **98**, 834–840 (1991). 2062516

[3] Bhanushali D (2016). Linking Retinal Microvasculature Features With Severity of Diabetic Retinopathy Using Optical Coherence Tomography Angiography. Investigative ophthalmology & visual science.

[4] Matsunaga DR (2015). Optical Coherence Tomography Angiography of Diabetic Retinopathy in Human Subjects. Ophthalmic surgery, lasers & imaging retina.

[5] Kim, K., Kim, E. S. & Yu, S. Y. Optical coherence tomography angiography analysis of foveal microvascular changes and inner retinal layer thinning in patients with diabetes. *The British journal of ophthalmology*, 10.1136/bjophthalmol-2017-311149 (2017). 10.1136/bjophthalmol-2017-311149 29259019

[6] Ting DSW (2017). Optical Coherence Tomographic Angiography in Type 2 Diabetes and Diabetic Retinopathy. JAMA ophthalmology.

[7] Tang FY (2017). Determinants of Quantitative Optical Coherence Tomography Angiography Metrics in Patients with Diabetes. Scientific reports.

[8] Kim M, Ha MJ, Choi SY, Park YH (2018). Choroidal vascularity index in type-2 diabetes analyzed by swept-source optical coherence tomography. Scientific reports.

[9] Guo J, She X, Liu X, Sun X (2017). Repeatability and Reproducibility of Foveal Avascular Zone Area Measurements Using AngioPlex Spectral Domain Optical Coherence Tomography Angiography in Healthy Subjects. Ophthalmologica. Journal international d’ophtalmologie. International journal of ophthalmology. Zeitschrift fur Augenheilkunde.

[10] Carpineto P (2016). Reproducibility and repeatability of foveal avascular zone measurements in healthy subjects by optical coherence tomography angiography. The British journal of ophthalmology.

[11] Shahlaee A, Pefkianaki M, Hsu J, Ho AC (2016). Measurement of Foveal Avascular Zone Dimensions and its Reliability in Healthy Eyes Using Optical Coherence Tomography Angiography. American journal of ophthalmology.

[12] Mastropasqua R (2017). Foveal avascular zone area and parafoveal vessel density measurements in different stages of diabetic retinopathy by optical coherence tomography angiography. International journal of ophthalmology.

[13] Cheung CY (2012). Retinal vascular geometry in Asian persons with diabetes and retinopathy. Journal of diabetes science and technology.

[14] Grauslund J (2010). Retinal vascular fractals and microvascular and macrovascular complications in type 1 diabetes. Ophthalmology.

[15] Savastano MC, Lumbroso B, Rispoli M (2015). *In Vivo* Characterization of Retinal Vascularization Morphology Using Optical Coherence Tomography Angiography. Retina (Philadelphia, Pa.).

[16] Chen Q (2017). Macular Vascular Fractal Dimension in the Deep Capillary Layer as an Early Indicator of Microvascular Loss for Retinopathy in Type 2 Diabetic Patients. Investigative ophthalmology & visual science.

[17] Coscas F (2016). Optical Coherence Tomography Angiography in Retinal Vein Occlusion: Evaluation of Superficial and Deep Capillary Plexa. American journal of ophthalmology.

[18] Muir ER, Renteria RC, Duong TQ (2012). Reduced ocular blood flow as an early indicator of diabetic retinopathy in a mouse model of diabetes. Investigative ophthalmology & visual science.

[19] Lutty GA, Cao J, McLeod DS (1997). Relationship of polymorphonuclear leukocytes to capillary dropout in the human diabetic choroid. The American journal of pathology.

[20] Nagaoka T (2004). Alteration of choroidal circulation in the foveal region in patients with type 2 diabetes. The British journal of ophthalmology.

[21] Say EAT (2017). Image Quality and Artifacts On Optical Coherence Tomography Angiography: Comparison of Pathologic and Paired Fellow Eyes in 65 Patients With Unilateral Choroidal Melanoma Treated With Plaque Radiotherapy. Retina (Philadelphia, Pa.).

[22] Spaide RF, Fujimoto JG, Waheed NK (2015). Image Artifacts In Optical Coherence Tomography Angiography. Retina (Philadelphia, Pa.).

[23] Tan CS (2016). Optical Coherence Tomography Angiography Evaluation of the Parafoveal Vasculature and Its Relationship With Ocular Factors. Investigative ophthalmology & visual science.

[24] Fan H (2017). Reduced Macular Vascular Density in Myopic Eyes. Chinese medical journal.

[25] Ho, J., Dans, K., You, Q., Nudleman, E. N. & Freeman, W. R. comparison of 3 mm × 3 mm versus 6 mm × 6 mm optical coherence tomography angiography scan sizes in the evaluation of non-proliferative diabetic retinopathy. *Retina (Philadelphia*, *Pa.)*, 10.1097/iae.0000000000001951 (2017). 10.1097/IAE.0000000000001951 PMC5963959 29190249

[26] Shimizu K, Kobayashi Y, Muraoka K (1981). Midperipheral fundus involvement in diabetic retinopathy. Ophthalmology.

[27] Warrow DJ, Hoang QV, Freund KB (2013). Pachychoroid pigment epitheliopathy. Retina (Philadelphia, Pa.).

[28] Grading diabetic retinopathy from stereoscopic color fundus photographs–an extension of the modified Airlie House classification. ETDRS report number 10. Early Treatment Diabetic Retinopathy Study Research Group. *Ophthalmology* **98**, 786–806 (1991). 2062513

[29] Stanga PE (2016). Swept-Source Optical Coherence Tomography Angio (Topcon Corp, Japan): Technology Review. Developments in ophthalmology.

[30] Fenner BJ (2017). Identification of imaging features that determine quality and repeatability of retinal capillary plexus density measurements in OCT angiography. The British journal of ophthalmology.

[31] Choi J (2017). Quantitative optical coherence tomography angiography of macular vascular structure and foveal avascular zone in glaucoma. PloS one.

[32] Khairallah M (2017). Optical Coherence Tomography Angiography in Patients with Behcet Uveitis. Retina (Philadelphia, Pa.).

[33] Agrawal R (2016). Choroidal Vascularity Index in Central Serous Chorioretinopathy. Retina (Philadelphia, Pa.).

[34] Perkins NJ, Schisterman EF (2006). The inconsistency of “optimal” cutpoints obtained using two criteria based on the receiver operating characteristic curve. American journal of epidemiology.

